# Supplementary material for: Enhanced spatial clustering of single-molecule localizations with graph neural networks
Source: Nat Commun. 2025 Nov 3;16:9693. doi: 10.1038/s41467-025-65557-7 (PMC12583556; doi:10.1038/s41467-025-65557-7)
Supplement: Supplementary file 1 — Supplementary Information [file 41467_2025_65557_MOESM1_ESM.pdf]

SUPPLEMENTARY INFORMATION

Enhanced Spatial Clustering of Single-Molecule Localizations with Graph Neural Networks

Jesús Pineda[1], Sergi Masó-Orriols[2,3], Montse Masoliver[2,3], Joan Bertran[2,3], Mattias Goksör[1], Giovanni Volpe[1*], Carlo Manzo[2,3*]

[1]Department of Physics, University of Gothenburg, Origovägen 6B, Gothenburg, SE-41296, Sweden.
[2]Facultat de Ciències, Tecnologia i Enginyeries, Universitat de Vic – Universitat Central de Catalunya (UVic-UCC), C. de la Laura, 13, Vic, 08500, Barcelona, Spain.
[3]Bioinformatics and Bioimaging, Institut de Recerca i Innovació en Ciències de la Vida i de la Salut a la Catalunya Central (IRIS-CC), Vic, 08500, Barcelona, Spain.

*Corresponding author(s). E-mail(s): giovanni.volpe@physics.gu.se; carlo.manzo@uvic.cat;

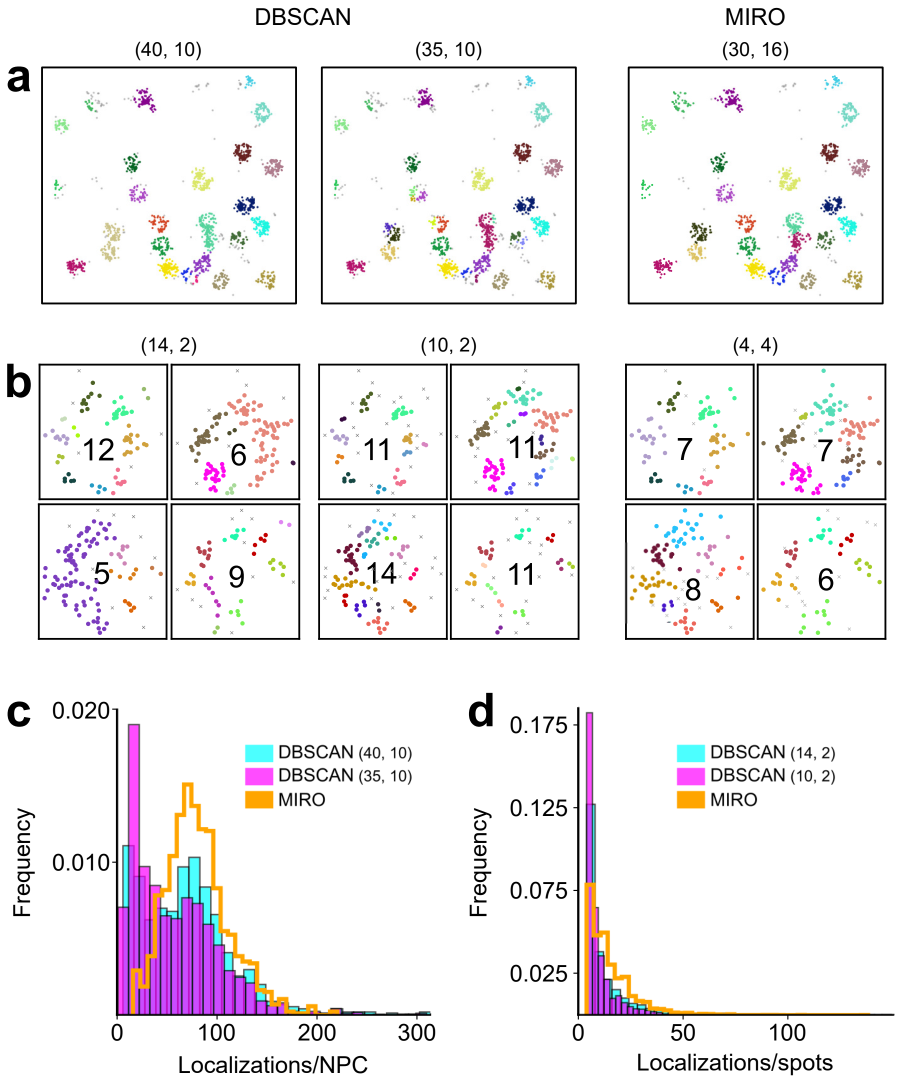

Supplementary Figure 1 Comparison of DBSCAN and MIRO performance in multi-scale analysis of nuclear pore complexes. (a) Examples of ring-like structures identified by DBSCAN (using two different parameter sets) and MIRO on a STORM localization map of nuclear pore complexes from Ref. [13]]. Localizations are colored according to the identified clusters, non-clustered localizations are shown in gray. (b) Examples of corner structures identified by DBSCAN (with two parameter sets) and MIRO within ring-like structures. Localizations are colored according to the identified corner and non-clustered localizations are indicated by gray crosses. Numbers denote the number of corners detected by each method. (c–d) Quantification of DBSCAN's clustering results at two scales: histograms of the number of localizations per nuclear pore complex (c) and per spot (d). Each color represents a different DBSCAN parameter set. The orange line in both panels shows the results obtained with MIRO. Source data are provided as a Source Data file.

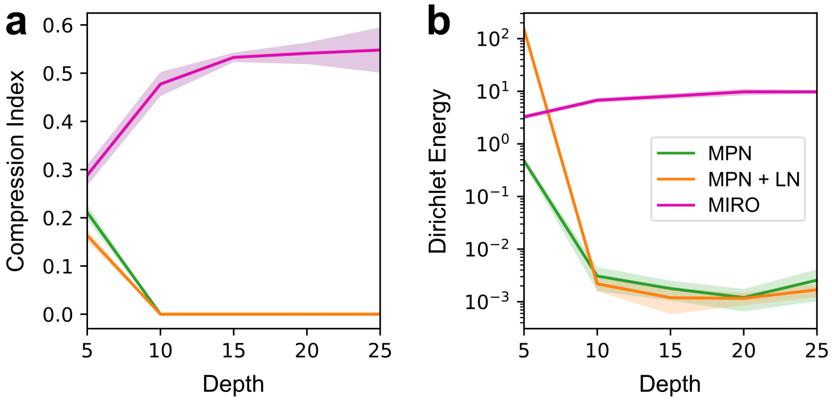

Supplementary Figure 2 Effect of the number of recurrent steps on clustering performance and oversmoothing. (**a**) Compression index and (**b**) Dirichlet energy computed for MIRO as a function of network depth (i.e., number of recurrent steps) on Scenario 6 with blinking. For comparison, results from a conventional message-passing network (MPN) — with and without layer normalization (LN) — are shown as the number of stacked layers increases. Each point represents the average over 5 independently trained models. Shaded areas indicate the standard deviation. Source data are provided as a Source Data file.

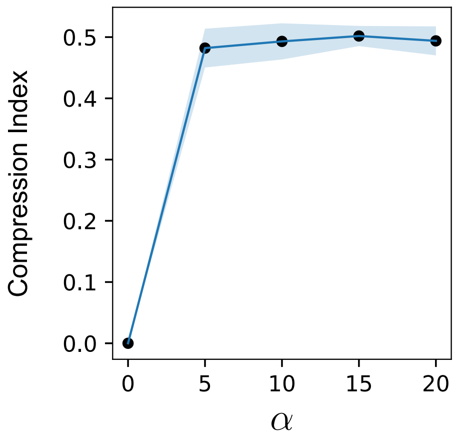

Supplementary Figure 3 Effect of the loss weight α on clustering performance. Compression index computed for MIRO as a function of the value of the loss weight α on Scenario 6 with blinking. Each point represents the average over 5 independently trained models. Shaded areas indicate the standard deviation. Source data are provided as a Source Data file.

Scenario	Method	ε	minPts
Scenario 2	MIRO	40.06	13
	DBSCAN[1]	45.00	7
Scenario 3	MIRO	15.58	12
	DBSCAN[2]	40.00	10
Scenario 4	MIRO	52.28	5
	DBSCAN[1]	50.00	4
Scenario 5	MIRO	26.00	13
	DBSCAN[1]	35.00	8
Scenario 6	MIRO	16.00	10
	DBSCAN[1]	30.00	6
Scenario 7	MIRO	67.97	8
	DBSCAN[1]	65.00	6
Scenario 8	MIRO	40.62	4
	DBSCAN[1]	50.00	4
Scenario 9	MIRO	13.00	6
	DBSCAN[1]	45.00	12
Scenario 10	MIRO	34.02	7
	DBSCAN[1]	45.00	7
Scenario 2 blinking	MIRO	20.01	38
	DBSCAN[1]	50.00	35
Scenario 3 blinking	MIRO	9.92	50
	DBSCAN[1]	45.00	50
Scenario 4 blinking	MIRO	31.45	23
	DBSCAN[1]	60.00	25
Scenario 5 blinking	MIRO	22.00	42
	DBSCAN[1]	35.00	34
Scenario 6 blinking	MIRO	31.34	63
	DBSCAN[1]	40.00	42
Scenario 7 blinking	MIRO	53.73	35
	DBSCAN[1]	65.00	32
Scenario 8 blinking	MIRO	20.00	30
	DBSCAN[1]	50.00	27
Scenario 9 blinking	MIRO	23.00	55
	DBSCAN[1]	45.00	50
Scenario 10 blinking	MIRO	20.71	38
	DBSCAN[1]	50.00	33

Supplementary Table 1 Summary of DBSCAN parameters used for each scenario of the benchmark.

[1] Values provided in the benchmark article.

[2] The parameter set used in this case yields significantly better scores than the one reported in the benchmark article ($\varepsilon = 30$, minPts = 10).

Scenario	Method	ε	minPts
C-shaped	MIRO<br>DBSCAN	115.20<br>85.76	15<br>5
Rings	MIRO<br>DBSCAN	115.20<br>115.20	15<br>12
Multi-shape (spots and ellipses)	MIRO<br>DBSCAN	26.00<br>—	5<br>—
Multi-shape (spots and rings)	MIRO<br>DBSCAN	64.00<br>—	5<br>—
Multi-shape (C-shaped and rings)	MIRO<br>DBSCAN	102.40<br>—	10<br>—
Integrin clusters	MIRO<br>DBSCAN	12<br>60.00	4<br>5
Multiscale rings (simulated)	MIRO<br>DBSCAN	56.96<br>76.80	7<br>4
Multi-scale spots (simulated)	MIRO<br>DBSCAN	35.20<br>42.24	4<br>4
Multi-scale rings (experimental)	MIRO<br>DBSCAN	30<br>40.00	16<br>10
Multi-scale spots (experimental)	MIRO<br>DBSCAN	4<br>10.00	4<br>2

Supplementary Table 2 Summary of DBSCAN parameters used for simulated and experimental scenarios.

Scenario	Method	ARI†	IoU	JI_c	$RMSRE_N$	$RMSE_{x,y}$	AMI	ARI_c	ARI
Scenario 2	MIRO	0.86 ± 0.02	0.80 ± 0.04	0.97 ± 0.04	0.18 ± 0.09	2.1 ± 0.2	0.87 ± 0.02	0.93 ± 0.04	0.87 ± 0.02
	DBSCAN	0.84 ± 0.03	0.74 ± 0.04	0.87 ± 0.09	0.3 ± 0.2	2.4 ± 0.4	0.86 ± 0.02	0.88 ± 0.06	0.85 ± 0.02
Scenario 3	MIRO	0.63 ± 0.06	0.75 ± 0.05	0.99 ± 0.02	0.16 ± 0.06	1.7 ± 0.2	0.86 ± 0.02	0.95 ± 0.03	0.90 ± 0.02
	DBSCAN	0.57 ± 0.07	0.70 ± 0.04	0.96 ± 0.06	0.23 ± 0.08	1.8 ± 0.2	0.85 ± 0.02	0.94 ± 0.04	0.89 ± 0.02
Scenario 4	MIRO	0.52 ± 0.13	0.44 ± 0.08	0.71 ± 0.10	0.32 ± 0.14	2.9 ± 0.4	0.68 ± 0.06	0.78 ± 0.12	0.69 ± 0.06
	DBSCAN	0.49 ± 0.12	0.39 ± 0.07	0.67 ± 0.11	0.39 ± 0.13	3.2 ± 0.4	0.65 ± 0.05	0.75 ± 0.11	0.67 ± 0.06
Scenario 5	MIRO	0.57 ± 0.02	0.62 ± 0.02	0.88 ± 0.03	0.56 ± 0.10	3.13 ± 0.12	0.657 ± 0.012	0.64 ± 0.03	0.57 ± 0.02
	DBSCAN	0.55 ± 0.03	0.56 ± 0.02	0.67 ± 0.04	1.1 ± 0.2	3.6 ± 0.2	0.637 ± 0.013	0.46 ± 0.04	0.56 ± 0.02
Scenario 6	MIRO	0.72 ± 0.03	0.67 ± 0.03	0.95 ± 0.04	0.22 ± 0.09	3.6 ± 0.4	0.783 ± 0.015	0.86 ± 0.04	0.76 ± 0.02
	DBSCAN	0.66 ± 0.03	0.57 ± 0.03	0.67 ± 0.07	0.5 ± 0.2	4.6 ± 0.7	0.73 ± 0.02	0.66 ± 0.07	0.73 ± 0.02
Scenario 7	MIRO	0.61 ± 0.04	0.44 ± 0.06	0.86 ± 0.08	0.5 ± 0.2	4.7 ± 0.6	0.65 ± 0.03	0.64 ± 0.06	0.58 ± 0.04
	DBSCAN	0.58 ± 0.04	0.34 ± 0.06	0.73 ± 0.09	0.7 ± 0.3	5.4 ± 0.6	0.61 ± 0.03	0.53 ± 0.07	0.53 ± 0.04
Scenario 8	MIRO	0.83 ± 0.04	0.72 ± 0.06	0.88 ± 0.08	0.16 ± 0.06	2.3 ± 0.3	0.85 ± 0.03	0.92 ± 0.04	0.83 ± 0.03
	DBSCAN	0.81 ± 0.04	0.65 ± 0.06	0.80 ± 0.11	0.3 ± 0.2	2.6 ± 0.3	0.83 ± 0.03	0.90 ± 0.07	0.80 ± 0.04
Scenario 9	MIRO	0.59 ± 0.03	0.69 ± 0.04	0.82 ± 0.07	0.5 ± 0.3	3.6 ± 0.5	0.67 ± 0.02	0.74 ± 0.07	0.61 ± 0.02
	DBSCAN	0.56 ± 0.03	0.58 ± 0.04	0.72 ± 0.09	0.8 ± 0.8	4.9 ± 0.8	0.62 ± 0.02	0.57 ± 0.08	0.58 ± 0.02
Scenario 10	MIRO	0.86 ± 0.03	0.80 ± 0.05	0.98 ± 0.03	0.17 ± 0.10	2.1 ± 0.3	0.87 ± 0.02	0.94 ± 0.03	0.86 ± 0.03
	DBSCAN	0.84 ± 0.03	0.74 ± 0.05	0.89 ± 0.08	0.27 ± 0.14	2.3 ± 0.4	0.86 ± 0.02	0.89 ± 0.05	0.84 ± 0.03

Supplementary Table 3 Summary of clustering metrics for non-blinking scenarios. Data represent mean ± standard deviation calculated over 47 fields of view. Training was performed over clusters contained in 3 fields of view.

Scenario	Method	ARI[†]	IoU	JI_c	$RMSRE_N$	$RMSE_{x,y}$	AMI	ARI_c	ARI
Scenario 2 blinking	MIRO	0.83 ± 0.03	0.71 ± 0.05	0.86 ± 0.07	0.26 ± 0.10	2.6 ± 0.3	0.83 ± 0.03	0.92 ± 0.04	0.75 ± 0.06
	DBSCAN	0.81 ± 0.04	0.68 ± 0.06	0.78 ± 0.11	0.5 ± 0.3	3.0 ± 0.5	0.81 ± 0.04	0.830 ± 0.099	0.73 ± 0.06
Scenario 3 blinking	MIRO	0.43 ± 0.13	0.57 ± 0.06	0.76 ± 0.10	0.31 ± 0.08	2.4 ± 0.2	0.71 ± 0.04	0.81 ± 0.11	0.72 ± 0.05
	DBSCAN	0.2 ± 0.2	0.49 ± 0.05	0.70 ± 0.11	0.6 ± 0.2	2.9 ± 0.3	0.68 ± 0.04	0.80 ± 0.11	0.69 ± 0.05
Scenario 4 blinking	MIRO	0.67 ± 0.10	0.31 ± 0.07	0.34 ± 0.08	0.6 ± 0.2	3.5 ± 0.5	0.47 ± 0.08	0.5 ± 0.2	0.35 ± 0.10
	DBSCAN	0.56 ± 0.13	0.27 ± 0.06	0.30 ± 0.09	0.8 ± 0.4	3.6 ± 0.6	0.45 ± 0.08	0.4 ± 0.2	0.34 ± 0.10
Scenario 5 blinking	MIRO	0.59 ± 0.02	0.56 ± 0.02	0.70 ± 0.04	0.78 ± 0.13	3.71 ± 0.14	0.62 ± 0.02	0.50 ± 0.05	0.37 ± 0.03
	DBSCAN	0.56 ± 0.03	0.54 ± 0.03	0.56 ± 0.05	1.3 ± 0.2	3.92 ± 0.15	0.60 ± 0.02	0.28 ± 0.05	0.42 ± 0.03
Scenario 6 blinking	MIRO	0.66 ± 0.04	0.66 ± 0.03	0.89 ± 0.06	0.40 ± 0.12	4.3 ± 0.5	0.74 ± 0.02	0.84 ± 0.05	0.65 ± 0.03
	DBSCAN	0.65 ± 0.03	0.61 ± 0.03	0.72 ± 0.09	0.6 ± 0.2	4.8 ± 0.5	0.70 ± 0.02	0.65 ± 0.06	0.66 ± 0.03
Scenario 7 blinking	MIRO	0.65 ± 0.04	0.43 ± 0.07	0.70 ± 0.09	0.5 ± 0.2	5.2 ± 0.4	0.65 ± 0.04	0.667 ± 0.095	0.45 ± 0.08
	DBSCAN	0.64 ± 0.05	0.34 ± 0.06	0.629 ± 0.095	0.6 ± 0.3	5.1 ± 0.7	0.62 ± 0.03	0.51 ± 0.08	0.45 ± 0.06
Scenario 8 blinking	MIRO	0.84 ± 0.04	0.62 ± 0.06	0.57 ± 0.07	0.28 ± 0.13	2.6 ± 0.4	0.77 ± 0.05	0.81 ± 0.08	0.65 ± 0.08
	DBSCAN	0.82 ± 0.05	0.59 ± 0.05	0.52 ± 0.05	0.4 ± 0.2	2.9 ± 0.6	0.76 ± 0.04	0.74 ± 0.08	0.64 ± 0.06
Scenario 9 blinking	MIRO	0.60 ± 0.04	0.64 ± 0.03	0.63 ± 0.07	0.7 ± 0.3	4.4 ± 0.7	0.64 ± 0.02	0.69 ± 0.07	0.56 ± 0.03
	DBSCAN	0.60 ± 0.04	0.60 ± 0.04	0.56 ± 0.09	0.9 ± 0.6	5.2 ± 0.9	0.61 ± 0.03	0.56 ± 0.11	0.55 ± 0.04
Scenario 10 blinking	MIRO	0.84 ± 0.04	0.72 ± 0.05	0.86 ± 0.07	0.22 ± 0.08	2.5 ± 0.3	0.83 ± 0.03	0.91 ± 0.04	0.75 ± 0.06
	DBSCAN	0.82 ± 0.03	0.67 ± 0.06	0.78 ± 0.09	0.4 ± 0.2	2.9 ± 0.4	0.81 ± 0.04	0.85 ± 0.06	0.72 ± 0.07

Supplementary Table 4 Summary of clustering metrics for blinking scenarios. Data represent mean ± standard deviation calculated over 47 fields of view. Training was performed over clusters contained in 3 fields of view.

Scenario	N	$\mathrm{ARI}^{\dagger}$	IoU	$\mathrm{JI_c}$	RMSRE_N	$\mathrm{RMSE}_{x,y}$	AMI	$\mathrm{ARI_c}$	ARI
Scenario 5	1	0.560 ± 0.007	0.614 ± 0.005	0.882 ± 0.008	0.54 ± 0.04	3.16 ± 0.05	0.658 ± 0.004	0.62 ± 0.03	0.576 ± 0.011
	300	0.566 ± 0.005	0.612 ± 0.008	0.870 ± 0.012	0.57 ± 0.04	3.16 ± 0.03	0.654 ± 0.005	0.64 ± 0.03	0.560 ± 0.012
Scenario 6	1	0.726 ± 0.009	0.662 ± 0.013	0.94 ± 0.02	0.24 ± 0.02	3.6 ± 0.2	0.788 ± 0.004	0.87 ± 0.02	0.752 ± 0.004
	60	0.724 ± 0.005	0.680 ± 0.010	0.94 ± 0.02	0.21 ± 0.02	3.59 ± 0.11	0.784 ± 0.005	0.864 ± 0.009	0.760 ± 0.007

Supplementary Table 5 Performance metrics comparing single-sample and full-dataset settings for representative scenarios. Each value is reported as mean ± standard deviation over 5 independently trained models.

Scenario	Method	$\mathrm{ARI}^{\dagger}$	IoU	$\mathrm{JI_c}$	RMSRE_N	$\mathrm{RMSE}_{x,y}$	AMI	$\mathrm{ARI_c}$	ARI
Scenario 5	MIRO	0.57 ± 0.02	0.62 ± 0.02	0.88 ± 0.03	0.56 ± 0.10	3.13 ± 0.12	0.657 ± 0.012	0.64 ± 0.03	0.57 ± 0.02
	MAGIK-S	0.47 ± 0.03	0.54 ± 0.02	0.58 ± 0.04	1.048 ± 0.098	3.9 ± 0.2	0.615 ± 0.013	0.57 ± 0.05	0.49 ± 0.02
	DBSCAN	0.55 ± 0.03	0.56 ± 0.02	0.67 ± 0.04	1.1 ± 0.2	3.6 ± 0.2	0.637 ± 0.013	0.46 ± 0.04	0.56 ± 0.02
Scenario 6	MIRO	0.72 ± 0.03	0.67 ± 0.03	0.95 ± 0.04	0.22 ± 0.09	3.6 ± 0.4	0.783 ± 0.015	0.86 ± 0.04	0.76 ± 0.02
	MAGIK-S	0.64 ± 0.04	0.60 ± 0.03	0.74 ± 0.06	0.22 ± 0.04	4.8 ± 0.5	0.75 ± 0.02	0.78 ± 0.04	0.74 ± 0.02
	DBSCAN	0.66 ± 0.03	0.57 ± 0.03	0.67 ± 0.07	0.5 ± 0.2	4.6 ± 0.7	0.73 ± 0.02	0.66 ± 0.07	0.73 ± 0.02

Supplementary Table 6 Performance metrics comparing MIRO with a supervised GNN-based clustering method (MAGIK-S) and DBSCAN for representative scenarios. Data represent mean ± standard deviation calculated over 47 fields of view. Training was performed over clusters contained in 3 fields of view.

Scenario	K	α	β	hidden size	δ	k^*
Scenario 2	12	10	—	256	0.2	—
Scenario 3	12	10	—	256	0.2	—
Scenario 4	12	10	—	256	0.2	—
Scenario 5	15	10	—	256	0.1	—
Scenario 6	15	10	—	256	0.2	—
Scenario 7	12	10	—	256	0.2	—
Scenario 8	12	10	—	256	0.2	—
Scenario 9	20	10	—	256	0.1	—
Scenario 10	12	10	—	256	0.1	—
C-shaped	20	10	—	256	0.1	—
Rings	20	10	—	256	0.1	—
Scenario 2 blinking	25	10	—	256	0.1	—
Scenario 3 blinking	15	10	—	256	0.1	—
Scenario 4 blinking	25	10	—	256	0.1	—
Scenario 5 blinking	10	10	—	256	0.1	—
Scenario 6 blinking	15	10	—	256	0.1	—
Scenario 7 blinking	15	10	—	256	0.1	—
Scenario 8 blinking	20	10	—	256	0.1	—
Scenario 9 blinking	25	10	—	256	0.1	—
Scenario 10 blinking	15	10	—	256	0.1	—
Multi-shape (spots and ellipses)	20	10	0.1	256	0.2	—
Multi-shape (spots and rings)	20	10	0.05	256	0.2	—
Multi-shape (C-shaped and rings)	20	10	0.05	256	0.2	—
Integrin clusters	20	10	—	256	0.2	—
Multi-scale rings (simulated)	30	10	—	256	0.2	15
Multi-scale rings (experimental)	30	10	—	256	0.2	15

Supplementary Table 7 Summary of hyperparameters used for the different scenarios.

Hidden Size	$ARI^{\dagger}$	IoU	JI_c	$RMSRE_N$	$RMSE_{x,y}$	AMI	ARI_c	ARI	F1
64	0.835 ± 0.004	0.695 ± 0.008	0.878 ± 0.009	0.309 ± 0.010	0.0730 ± 0.0015	0.840 ± 0.004	0.808 ± 0.008	0.727 ± 0.008	0.915 ± 0.007
128	0.851 ± 0.005	0.731 ± 0.004	0.890 ± 0.008	0.292 ± 0.013	0.0687 ± 0.0013	0.863 ± 0.002	0.850 ± 0.003	0.768 ± 0.002	0.923 ± 0.007
256	0.854 ± 0.006	0.745 ± 0.012	0.917 ± 0.010	0.227 ± 0.015	0.064 ± 0.002	0.864 ± 0.006	0.861 ± 0.012	0.7764 ± 0.0098	0.929 ± 0.004
512	0.852 ± 0.004	0.739 ± 0.006	0.920 ± 0.003	0.228 ± 0.013	0.0634 ± 0.0013	0.862 ± 0.004	0.856 ± 0.009	0.773 ± 0.007	0.927 ± 0.003

Supplementary Table 8 Summary of performance metrics obtained for different hidden sizes on the dataset containing mixed shapes (spots and ellipses). Each value represents the average (± the standard deviation) over 5 independently trained models.

α	β	ARI[†]	IoU	JI_c	$RMSRE_N$	$RMSE_{x,y}$	AMI	ARI_c	ARI	F1
0	0.1	0.861 ± 0.003	0.748 ± 0.002	0.910 ± 0.008	0.26 ± 0.03	0.0632 ± 0.0012	0.868 ± 0.002	0.857 ± 0.003	0.7796 ± 0.0014	0.9335 ± 0.0014
5	0.1	0.8606 ± 0.0014	0.749 ± 0.006	0.913 ± 0.008	0.24 ± 0.02	0.0625 ± 0.0008	0.869 ± 0.002	0.863 ± 0.009	0.783 ± 0.005	0.9333 ± 0.0006
10	0.1	0.858 ± 0.003	0.747 ± 0.008	0.9086 ± 0.0096	0.25 ± 0.03	0.064 ± 0.002	0.869 ± 0.003	0.861 ± 0.009	0.782 ± 0.006	0.931 ± 0.003
15	0.1	0.855 ± 0.006	0.746 ± 0.006	0.916 ± 0.003	0.231 ± 0.008	0.0647 ± 0.0014	0.866 ± 0.004	0.859 ± 0.009	0.781 ± 0.006	0.929 ± 0.005
20	0.1	0.856 ± 0.004	0.744 ± 0.009	0.909 ± 0.007	0.243 ± 0.007	0.06422 ± 0.00095	0.866 ± 0.005	0.857 ± 0.011	0.779 ± 0.008	0.929 ± 0.003
10	0.05	0.855 ± 0.003	0.749 ± 0.004	0.915 ± 0.005	0.239 ± 0.009	0.0648 ± 0.0010	0.868 ± 0.002	0.864 ± 0.005	0.783 ± 0.003	0.930 ± 0.003
10	0.075	0.855 ± 0.003	0.743 ± 0.008	0.914 ± 0.006	0.242 ± 0.015	0.0642 ± 0.0009	0.865 ± 0.003	0.857 ± 0.008	0.778 ± 0.006	0.931 ± 0.002
10	0.1	0.855 ± 0.004	0.746 ± 0.006	0.914 ± 0.005	0.239 ± 0.015	0.0641 ± 0.0013	0.866 ± 0.003	0.862 ± 0.007	0.781 ± 0.004	0.928 ± 0.003
10	0.125	0.854 ± 0.003	0.742 ± 0.003	0.913 ± 0.006	0.245 ± 0.014	0.0650 ± 0.0006	0.8648 ± 0.0009	0.857 ± 0.003	0.778 ± 0.002	0.927 ± 0.003
10	0.15	0.857 ± 0.003	0.751 ± 0.005	0.914 ± 0.009	0.25 ± 0.02	0.064 ± 0.002	0.869 ± 0.002	0.866 ± 0.006	0.783 ± 0.003	0.931 ± 0.002
10	0.175	0.859 ± 0.002	0.747 ± 0.005	0.914 ± 0.006	0.245 ± 0.009	0.0642 ± 0.0009	0.868 ± 0.003	0.863 ± 0.008	0.782 ± 0.004	0.929 ± 0.004
10	0.2	0.859 ± 0.002	0.750 ± 0.006	0.919 ± 0.004	0.233 ± 0.004	0.0629 ± 0.0006	0.868 ± 0.002	0.865 ± 0.005	0.783 ± 0.004	0.932 ± 0.002
10	0.5	0.861 ± 0.003	0.750 ± 0.003	0.909 ± 0.007	0.25 ± 0.02	0.0622 ± 0.0006	0.870 ± 0.002	0.864 ± 0.003	0.784 ± 0.002	0.933 ± 0.003

Supplementary Table 9 Summary of performance metrics obtained for different values of the loss weights α and β on the dataset containing mixed shapes (spots and ellipses). Each value represents the average ± the standard deviation over 5 independently trained models.
